# A Woman With Solar Urticaria and Heat Urticaria: A Unique Presentation of an Individual With Multiple Physical Urticarias

**DOI:** 10.7759/cureus.16950

**Published:** 2021-08-06

**Authors:** Kyra L Diehl, Christof Erickson, Antoanella Calame, Philip R Cohen

**Affiliations:** 1 Osteopathic Medicine, Western University of Health Sciences, Pomona, USA; 2 Dermatology, Compass Dermatopathology, San Diego, USA; 3 Dermatology/Dermatopathology, Compass Dermatopathology, San Diego, USA; 4 Dermatology, Scripps Memorial Hospital, La Jolla, USA; 5 Dermatology, San Diego Family Dermatology, National City, USA

**Keywords:** angioedema, erythematous, heat, histamine, hive, mast, urticaria, photosensitivity, solar, wheal

## Abstract

Urticaria is a common group of dermatologic disorders characterized by hives. Solar urticaria and heat urticaria are two rare types of chronic inducible urticarias. Solar urticaria is triggered by exposure to sunlight or ultraviolet radiation. Heat urticaria is triggered by exposure or contact with a heat stimulus. A 63-year-old woman is described who has both solar urticaria and heat urticaria and the features of these chronic inducible urticarias are reviewed. The woman presented with urticarial lesions that appeared both after exposure to the sun and after cooking at a stove. Additional history revealed she was previously diagnosed with diabetes, hypertension, and thyroid disease. After sun exposure, a punch biopsy of both the affected skin, as well as the normal-appearing skin, was done. Correlation of the clinical history, cutaneous examination, and biopsy examination confirmed the diagnosis of solar urticaria. Treatment of the patient’s urticarias included histamine 1 (H1) and histamine 2 (H2) antihistamines. Her symptoms resolved and did not recur provided that she took the medication as prescribed. Management of chronic urticaria includes not only treatment of the current episode but also prevention of future recurring urticarial lesions. In addition to antihistamines, treatment may include omalizumab (Xolair®) injections for persistent urticaria.

## Introduction

Solar urticaria is an IgE-mediated hypersensitivity. It presents as hives and is caused by ultraviolet radiation or, albeit less commonly, visible light (400 to 750 nanometers). Several classifications based on either the wavelength of ultraviolet radiation or associated allergens have been proposed [1-3]. 

Heat urticaria results from contact with the responsible thermal source; mast cell activation and histamine release are postulated to be involved in the pathogenesis. Clinical presentations can include either immediate and localized lesions, immediate and generalized hives, or delayed onset of wheals [4-6]. 

A woman with not only solar urticaria but also heat urticaria is described. The salient features of heat and solar urticaria are summarized. An approach to the management of urticaria is summarized.

## Case presentation

**Figure 1:**
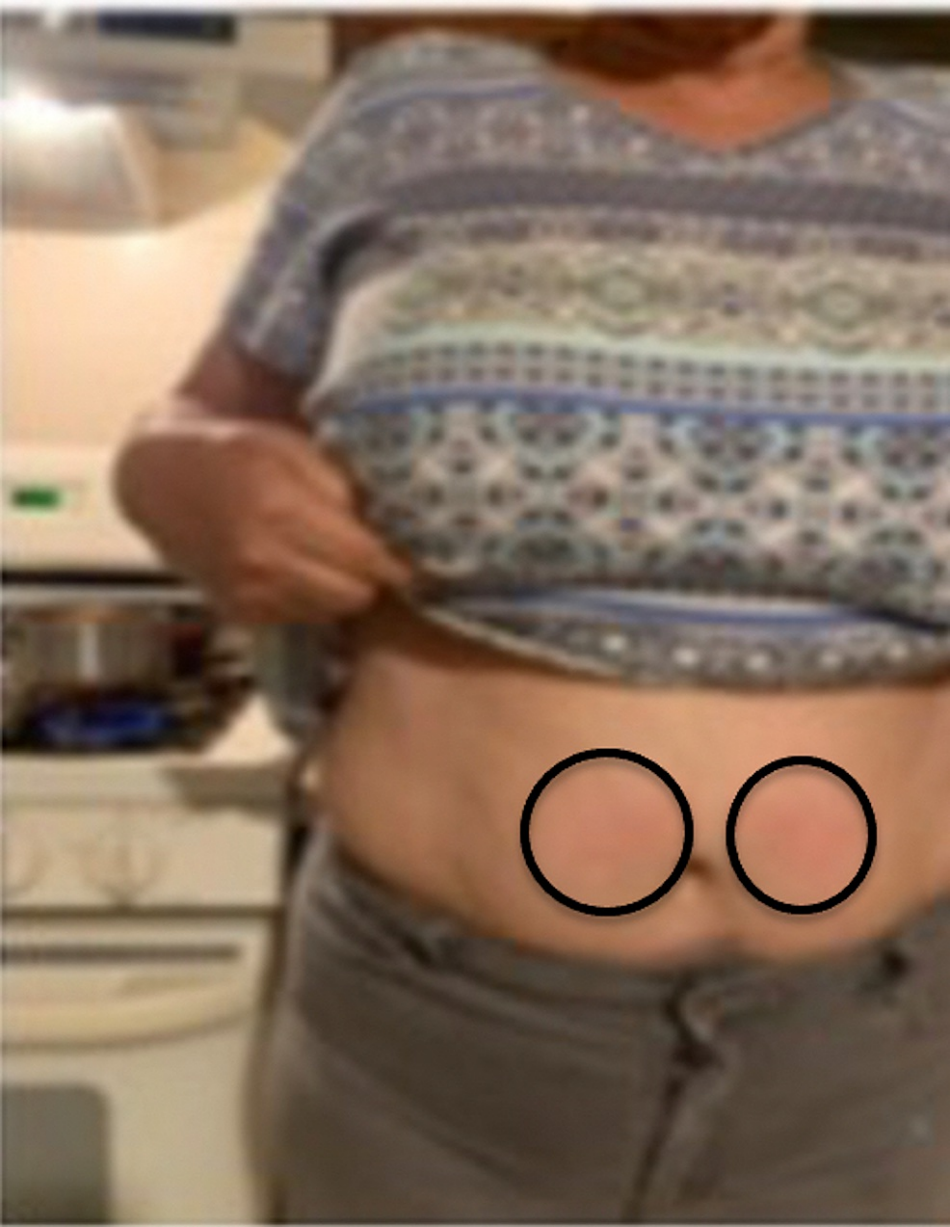
Clinical presentation of heat urticaria A 63-year-old woman presents with urticarial lesions on her abdomen after cooking at her stove. The clinical features of her heat urticaria – erythematous and pruritic periumbilical wheals – are shown (black ovals) on the frontal view of her abdomen

A 63-year-old woman with a past medical history of diabetes, hypertension, and hypothyroidism presented for evaluation of repeat episodes of an itchy rash on her body after exposure to the sun, as well as recurrent erythema on her abdomen that appeared each time she cooked at a stove. Both of her skin conditions had been present for at least three years. She provided a photograph of periumbilical erythematous hives on her abdomen that repeatedly developed when she would cook at the countertop stove (Figure 1). Her current medications included levothyroxine, losartan, and metoprolol. 

**Figure 2:**
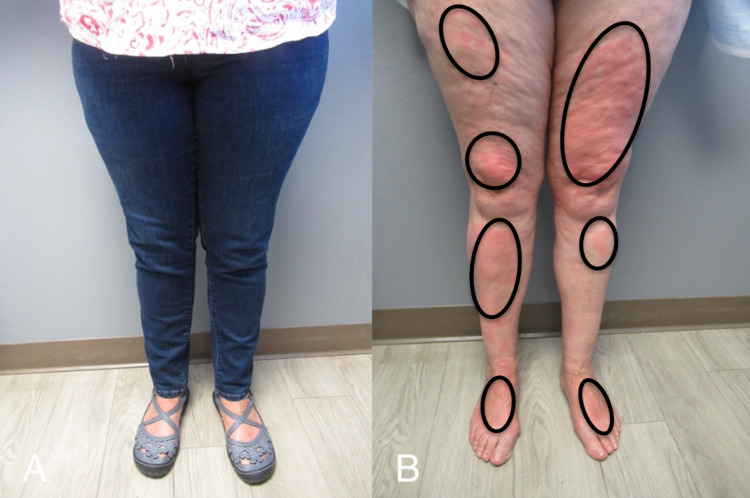
Clinical presentation of solar urticaria Urticarial lesions developed on the legs of a 63-year-old woman after standing outside in a sun-exposed parking lot for 15 minutes with her pants and sandals on (A). Her anterior legs and dorsal feet (B) demonstrate solar urticaria presenting as erythematous, pruritic wheals (black ovals).

**Figure 3:**
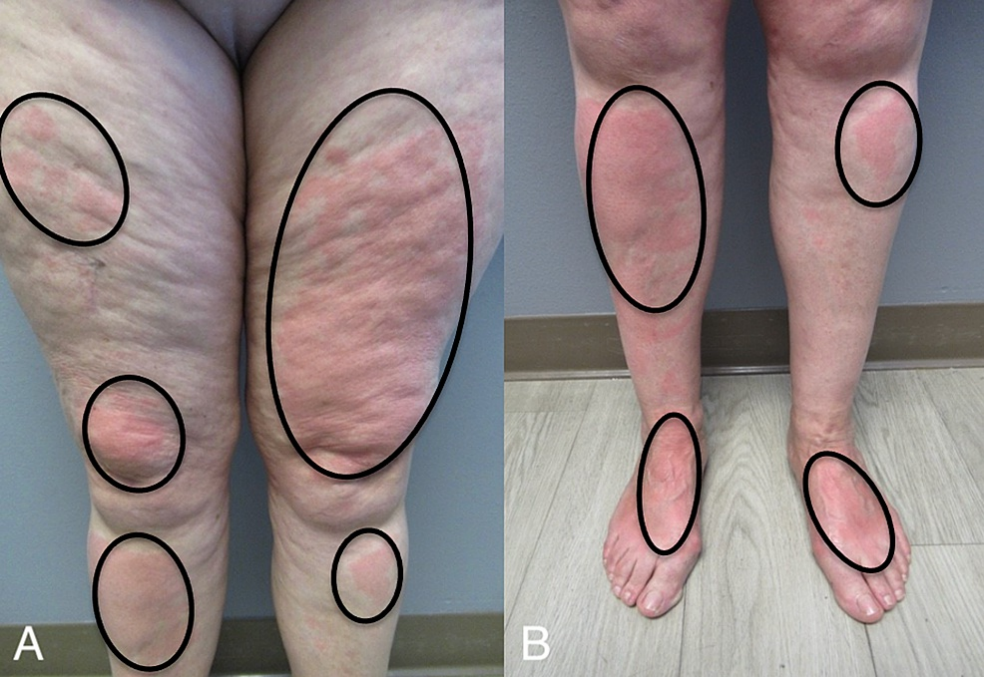
Solar urticaria appears as red itchy wheals Closer views of the anterior thighs (A), pretibial legs (A and B), distal legs (B), and dorsal feet (B) showed erythematous areas of urticaria (black ovals).

**Figure 4:**
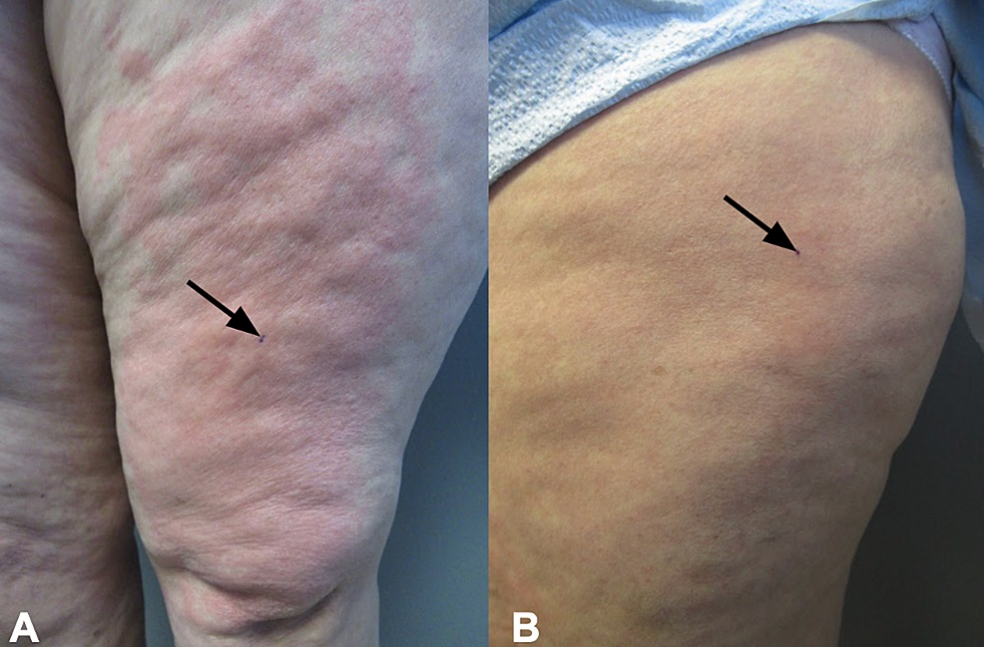
Skin biopsy sites of affected and normal-appearing skin from a woman with solar urticaria The lesional skin biopsy site (black arrow pointing to purple dot) was taken from affected erythematous skin of the left thigh (A). A biopsy of normal-appearing skin was taken from an area of flesh-colored skin (black arrow pointing to purple dot) on the left lateral thigh (B).

In addition, she would develop hives not only in sun-exposed but also in sun-covered areas after being outside. To demonstrate the lesions, prior to a follow-up visit she stood outside in the parking lot for 15 minutes with her pants and sandals on. Extremely pruritic, erythematous urticarial lesions developed on both covered and exposed areas of her legs and arms (Figure 2, 3). Skin biopsies from both an affected area on her left anterior thigh and a normal-appearing site on her left lateral thigh were performed (Figure 4).

**Figure 5:**
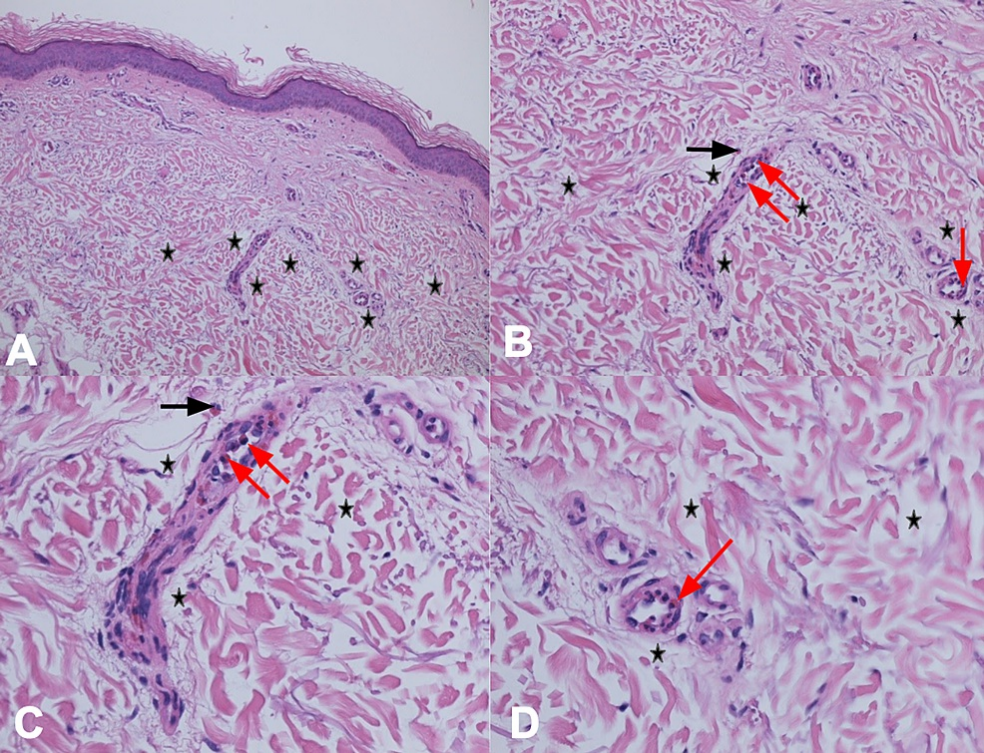
Pathology changes of solar urticaria Distant (A) and closer (B, C, and D) views of the lesional skin biopsy from the left thigh shows widening of the spaces between the collagen bundles (edema) in the dermis (black stars) and occasional eosinophils in both the edema (black arrow) and blood vessels (red arrows) in the dermis (hematoxylin and eosin: A, x 4; B, x 20; C, x 40; D, x 40).

**Figure 6:**
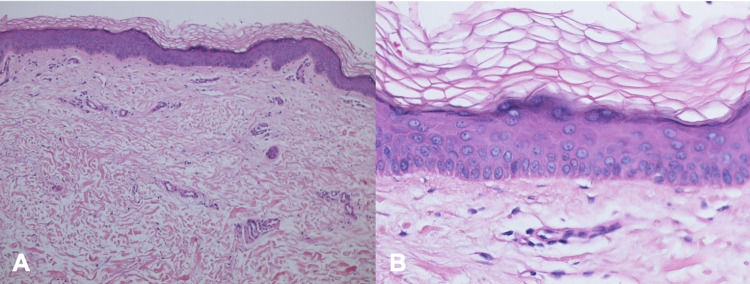
Microscopic features of normal-appearing skin Distant (A) and closer (B) views of the skin biopsy of normal-appearing skin for comparison to the lesional skin biopsy of solar urticaria. Neither edema nor eosinophils are observed in the dermis (hematoxylin and eosin: A, x 4; B, x 20).

Microscopic examination of the lesional skin biopsy showed widening of the spaces between the collagen bundles, consistent with edema. In addition, occasional eosinophils were present not only in the dermal blood vessels but also in the dermis (Figure 5). In contrast, the biopsy from the normal-appearing skin did not show any pathologic alteration; there was neither increased space between the collagen bundles nor eosinophils in the dermis (Figure 6). 

Laboratory studies were performed. These included a complete blood count, comprehensive metabolic panel, serum protein electrophoresis, thyroid-stimulating hormone, thyroxine, triiodothyronine as well as tests for complement components, DNA antibodies, HIV antibodies, rheumatoid factor, ribonucleic protein antibody, scleroderma-70 antibodies, Sjogren syndrome A antibody, Sjogren syndrome B antibody, Smith antibody, and thyroid antibodies (peroxidase). All of the studies were either negative or normal except for a blood urea nitrogen/creatinine ratio of 23 (normal, six to 22), creatinine level of 1.05 mg/dL (normal, 0.50-0.99 mg/dL), and thyroid peroxidase antibodies of 157 IU/mL (normal, less than nine IU/mL). 

Correlation of the clinical history, pathologic changes, and laboratory studies established not only a diagnosis of solar urticaria but also heat urticaria. Her initial management included histamine 1 (H1) and histamine 2 (H2) antihistamines, specifically fexofenadine 180 milligrams and famotidine 20 milligrams in the morning, and hydroxyzine 10 milligrams and famotidine 20 milligrams in the evenings. She tolerated this therapeutic regimen without any adverse effects. 

A follow-up visit showed that all symptoms and lesions resolved and did not recur even with occasional exposure to sunlight when she took the medications as prescribed. However, any variance of treatment resulted in recurrence of her urticaria, specifically when going outside. She continues to be managed on her current therapy without any additional flares of urticaria.

## Discussion

Urticaria is a common dermatologic condition characterized by pruritic, erythematous wheals; in addition, it can be accompanied by angioedema of the subcutaneous or interstitial tissue. Urticaria can be further classified into either acute or chronic types. Acute urticaria is characterized by lesions that occur for less than six weeks and commonly has precipitating triggers. Chronic urticaria is the persistence of lesions beyond six weeks and can be further classified into chronic idiopathic urticaria or chronic inducible urticaria [7,8].

The symptoms of chronic idiopathic (also referred to as spontaneous) urticaria occur with no known cause. In contrast, chronic inducible urticaria is triggered in response to a specific physical or environmental stimulus. These stimuli include cold, exercise, heat, light, pressure, and vibration [7,9]. 

Chronic inducible urticaria can be further classified into either physical or non-physical urticarias. Physical urticarias include not only solar urticaria and heat urticaria, but also cold urticaria, delayed-pressure urticaria, dermographism, exercise-induced urticaria, and vibratory urticaria. Non-physical urticarias include aquagenic urticaria, cholinergic urticaria, and contact urticaria [9]. 

Solar urticaria is a rare type of physical urticaria which is immunoglobulin E (IgE)-mediated. It typically presents with short-lasting, pruritic, erythematous patches and wheals that form approximately five to ten minutes after exposure to sunlight. Solar urticaria can also be associated with systemic manifestations, such as dizziness, headaches, nausea, syncope, wheezing, and rarely anaphylactic shock [1-3,10]. 

Solar urticaria mainly affects women and most patients present between the ages of 20 to 40 years. The symptoms associated with solar urticaria can vary in severity and duration depending on the length of light exposure, the intensity of light, and location. Direct light exposure is required; however, in some patients, symptoms may occur even when exposed to light while wearing clothing, such as our patient [1,2]. 

The pathogenesis of solar urticaria remains to be definitively established. The specific wavelengths of light (action spectrum) that elicit an urticarial reaction varies in each patient. However, most of the urticarial reactions occur following exposure to ultraviolet radiation ranging from 280-500 nanometers [1,2]. 

Investigators have proposed that the urticaria-triggering action spectra induces a cutaneous chromophore to absorb the light. The photoproduct that is created then acts as a photoallergen. Subsequently, IgE antibodies produced to the photoallergen bind to mast cells, resulting in histamine release [1,2]. 

Originally, in 1963, six types of solar urticaria were named by determining not only the amount of energy needed to elicit an urticarial reaction with an ultraviolet light meter but also whether an intradermal injection of the patient’s serum into their own forearm prior to irradiation of the site (passive transfer) or into the forearm of a non-solar urticaria volunteer after irradiation of the site (reverse passive transfer) resulted in urticaria. The nanometer range of the action spectrum was from 285 to 500: 285 to 320 (Type I), 320 to 400 (Type II), 400 to 500 (Type III and Type IV), 280 to 500 (Type V), and 400 (Type VI). Passive transfer was present in patients with Type I and Type IV solar urticaria and reverse passive transfer was only observed in individuals with Type I solar urticaria. Patients with Type V solar urticaria were sensitive to multiple diffuse bands of radiation. Patients with Type VI solar urticaria had photosensitivity due to protoporphyrinemia, and urticarial lesions occurred upon exposure to radiation at 400 nanometers, which corresponds to the absorption peak of protoporphyrin IX [11]. 

Subsequently, in 1989, solar urticaria was reclassified into two different types. Type I solar urticaria is caused by specific photoallergens present in solar urticaria patients against which IgE antibodies are directed. In contrast, Type II solar urticaria is caused by a nonspecific photoallergen - generated not only in patients with solar urticaria but also individuals without solar urticaria - that induces IgE-mediated immediate-type hypersensitivity [1,12]. 

Fixed solar urticaria is an additional distinctive form of solar urticaria. It is characterized by repeated episodes of wheals that are localized to only specific skin sites for that individual. There are not any associated systemic symptoms in patients with fixed solar urticaria [2]. 

Heat urticaria is a rare form of chronic inducible urticaria that is characterized by pruritic, erythematous, well-demarcated wheals that form after heat exposure. Associated systemic symptoms, such as headaches, nausea, tachycardia, vomiting, and weakness may occur. Heat urticaria most commonly occurs in women and the average age of onset is 20 to 45 years. The most common triggers are contact with warm objects or foods, exposure to the sun, hot air, and warm baths. The average reported threshold temperature for symptoms is 44 degrees Celsius [5,13]. 

The pathogenesis of heat urticaria involves the activation of mast cells. Subsequently, there is a release of inflammatory mediators, such as histamine. However, the stimulus that causes the activation of mast cells remains to be determined [4,6]. 

Heat urticaria has been classified as either immediate onset or delayed onset. In immediate heat urticaria, the lesions are pruritic and appear within minutes and resolve within one to three hours. Heat urticaria can be either localized or generalized onset; in localized immediate heat urticaria, the lesions are limited to areas of the skin that came in direct contact with or was exposed to the heat stimulus. In generalized immediate heat urticaria, the lesions are diffuse and not limited to areas in contact with the heat stimulus [5,6]. 

Delayed onset heat urticaria is characterized by wheals that have a burning sensation and occur 30 minutes to two hours after heat contact and resolve within 12 to 14 hours. Delayed onset heat urticaria is a familial condition with an autosomal dominant inheritance and its initial observation is usually in children. In contrast, immediate heat urticaria typically presents in adult women and is not inherited [5,6]. 

The woman reported in this paper has both solar urticaria and heat urticaria. This is a unique presentation of multiple physical urticarias. In a comprehensive review of heat urticaria, the investigators noted 8% (four of 50) of the patients with heat urticaria also experienced either cold urticaria or dermographism. They also observed that occasional episodes of generalized lesions of heat urticaria occurred after exposure to a general heat environment such as sun exposure in 50% (24 of 48) of the patients; however, the researchers were able to exclude solar urticaria in 12 of these patients when additional testing to ultraviolet A, ultraviolet B, and visible light did not result in urticarial lesions [5].

The first-line treatment for urticaria is a non-sedating H1 antihistamine, such as bilastine, desloratadine, fexofenadine, levocetirizine, and loratadine. Antihistamines block the effect of histamine on either H1 or H2 receptors of endothelial or sensory nerve cells, which reduces the effect of mast cell mediators on target organs. If symptoms persist, the dosage of the H1 antihistamine can be increased by up to four times the standard dose [14,15]. 

After one to four weeks, if the symptoms are not controlled, an H2 antihistamine such as cimetidine or famotidine can be added. Another option would be to add a leukotriene antagonist, such as montelukast, to block mast cell release. Our patient was concurrently treated with both an H1 antihistamine and an H2 antihistamine; a sedating H1 antihistamine (hydroxyzine) was prescribed in the evening to enable sleep [14,16]. 

The management of chronic idiopathic urticaria may include corticosteroids. In addition to oral corticosteroids, other systemic agents such as dapsone or methotrexate can be used in severe cases of acute urticaria or chronic idiopathic urticaria. Intramuscular corticosteroids, such as triamcinolone acetonide, can be used to control episodic flares of urticaria; the injection should be made deep into a muscle, such as the gluteus maximus muscle located in the upper lateral quadrant of the buttock. However, the usage of oral or injected corticosteroids is not recommended for long-term use due to adverse effects; therefore, they should be tapered once symptoms are controlled [8,15]. 

More recently, omalizumab has been used for patients with chronic urticaria. Omalizumab is a monoclonal anti-IgE antibody that has been shown to be extremely effective for patients with chronic idiopathic urticaria, cholinergic urticaria, cold urticaria, and solar urticaria. Omalizumab is administered as a subcutaneous injection that is given once every four weeks [15,16].

## Conclusions

Physical urticaria is a class of chronic inducible urticaria. Our patient had two subtypes of physical urticaria: solar urticaria and heat urticaria. Although individuals may develop multiple types of physical urticaria, we are not aware of another individual with this unique combination of physical urticarias. She was successfully managed with H1 and H2 antihistamines with clearing of the urticaria and prevention of new lesions as long as she maintained the recommended treatment. The diagnosis of a physical urticaria can often be suspected based on the clinical history; however, skin biopsy or laboratory testing or both may be helpful in establishing the diagnosis.

## References

[1] Botto NC, Warshaw EM (2008). Solar urticaria. J Am Acad Dermatol.

[2] Goetze S, Elsner P (2015). Solar urticaria. J Dtsch Dermatol Ges.

[3] Komarow HD, Eisch AR, Young M, Nelson C, Metcalfe DD (2015). Solar urticaria. J Allergy Clin Immunol Pract.

[4] Chung HS, Lee KH, Ro JY (1996). Heat contact urticaria--a case report. Yonsei Med J.

[5] Pezzolo E, Peroni A, Gisondi P, Girolomoni G (2016). Heat urticaria: a revision of published cases with an update on classification and management. Br J Dermatol.

[6] White F, Cobos G, Soter NA (2017). Local heat urticaria. Dermatol Online J.

[7] Antia C, Baquerizo K, Korman A, Bernstein JA, Alikhan A (2018). Urticaria: a comprehensive review: epidemiology, diagnosis, and work-up. J Am Acad Dermatol.

[8] Schaefer P (2017). Acute and chronic urticaria: evaluation and treatment. Am Fam Physician.

[9] Pozderac I, Lugović-Mihić L, Artuković M, Stipić-Marković A, Kuna M, Ferček I (2020). Chronic inducible urticaria: classification and prominent features of physical and non-physical types. Acta Dermatovenerol Alp Pannonica Adriat.

[10] Kieselova K, Santiago F, Henrique M (2019). Incapacitating solar urticaria: successful treatment with omalizumab. An Bras Dermatol.

[11] Harber LC, Holloway RM, Wheatley VR, Baer RL (1963). Immunologic and biophysical studies in solar urticaria. J Invest Dermatol.

[12] Leenutaphong V, Holzle E, Plewig G (1989). Pathogenesis and classification of solar urticaria: a new concept. J Am Acad Dermatol.

[13] Bonnekoh H, Terhorst-Molawi D, Buttgereit T, Maurer M, Altrichter S (2020). Treatment of severe heat urticaria with omalizumab - report of a case and review of the literature. J Eur Acad Dermatol Venereol.

[14] Kröpfl L, Maurer M, Zuberbier T (2010). Treatment strategies in urticaria. Expert Opin Pharmacother.

[15] Kulthanan K, Tuchinda P, Chularojanamontri L (2016). Clinical practice guideline for diagnosis and management of urticaria. Asian Pac J Allergy Immunol.

[16] Zuberbier T, Aberer W, Asero R (2018). The EAACI/GA²LEN/EDF/WAO guideline for the definition, classification, diagnosis and management of urticaria. Allergy.

